# Differential treatment of individuals with mental health conditions in high-consequence decision-making: a comparison of policy on advance directives and assisted suicide in three European countries

**DOI:** 10.3389/fpsyt.2025.1616011

**Published:** 2025-08-15

**Authors:** Sophie Gloeckler, Matthé Scholten, Nikola Biller-Andorno

**Affiliations:** ^1^ Institute for Biomedical Ethics and History of Medicine, University of Zürich, Zürich, Switzerland; ^2^ Institute for Medical Ethics and History of Medicine, Ruhr University Bochum, Bochum, Germany

**Keywords:** assisted suicide, aid in dying, advance directives, psychiatry, coercion, discrimination, policy, autonomy

## Abstract

While both assisted suicide and advance directives relate to potentially high-consequence decision-making, the procedures for assisted suicide requests and expressing preferences through an advance directive typically place such processes outside of acute, emergency scenarios. These contexts allow for the decisions to be well-considered. As such, assisted suicide for individuals with mental health conditions and psychiatric advance directives present two valuable cases to examine how well-considered preferences with potentially high consequences are treated. The following study compares policies regarding assisted suicide and advance directives in Switzerland, the Netherlands, and Germany, highlighting policy distinctions between psychiatric and nonpsychiatric cases. By analyzing the implications of these various regulatory frameworks, the paper’s aim is to support well-founded legal and clinical practice across jurisdictions with attention to potentially discriminatory practices. All three jurisdictions create conditions whereby those with mental health conditions can, theoretically, access assisted suicide. In all three jurisdictions, treatment refusals expressed in advance directives for non-psychiatric care are binding, even if such refusals may be life-limiting, but the three jurisdictions handle the risk a person can assume through an advance directive in psychiatric cases quite differently from one another. The overarching regulatory differences found can be summarized as 1) a high degree of deference to clinician judgment in Switzerland, 2) arguments founded on the clinician’s duties to patients in the Netherlands, and 3) recognition of inviolable rights that apply uniformly to all in Germany; each has different implications when it comes to the rights of those with mental health conditions. Countries can use these findings toward a critical review of the policies that define respect for well-considered, high-consequence decisions, avoiding unjustified differential treatment of those with mental health conditions.

## 1 Introduction

Valid requests for assisted suicide and legitimate advance directives both require decision-making capacity (see glossary at the end of the article for definitions of key terms as used). Many people with mental health conditions retain decision-making capacity or have periods of stability where they have decision-making capacity (1–4). High-consequence decision-making refers to choices that carry profound and potentially irreversible outcomes for an individual’s health or well-being. Both requests for assisted suicide and advance directives can involve high-consequence decision-making: the first regards the choice to end one’s life and the second can be used to establish life-limiting treatment refusals. While both assisted suicide and advance directives relate to potentially high-consequence decision-making, the procedures for assisted suicide and advance directives typically place such processes outside of acute, emergency scenarios. As such, the decisions may be demonstrably well-considered by the individuals making them.

Assisted suicide for individuals with mental health conditions and psychiatric advance directives, therefore, present two valuable cases to examine how well-considered preferences with potentially high consequences are treated. Removed from high-risk emergency settings that complicate an evaluation of decision-making capacity and introduce justification for coercive measures, these cases are especially illuminating. If decision-making capacity has been established regarding the relevant high-consequence decision, how should differential treatment of that decision in psychiatric cases be understood and can such distinction be justified? **Table 1** presents a general framing of foundational equity questions for assisted suicide and advance directives in psychiatric cases.

**Table 1:** Foundational equity questions for assisted suicide and advance directives in psychiatric cases.

Practice	The question of psychiatric cases	The question of differential treatment
Assisted Suicide	If those with unbearable suffering have recourse to assisted suicide, should those whose unbearable suffering is due to psychiatric conditions have similar recourse?	If not, which aspects of decision-making in the face of unbearable psychiatric suffering are qualitatively different enough to disqualify those with psychiatric conditions?
Psychiatric Advance Directives	If someone has the right to use an advance directive (AD) to refuse lifesaving or life-sustaining treatment, should someone with psychiatric conditions similarly have the right to use an AD to refuse unwanted, potentially lifesaving, interventions – psychiatric or otherwise?	If not, which aspects of the risk of harm to self in case of psychiatric conditions is qualitatively different enough to warrant the distinctions?

Underlying this discussion is the question of whether those with mental health conditions face differential treatment when it comes to respect for autonomy and the right to assume personal risk (5–7). Some have questioned whether there are discriminatory practices when it comes to policy around assisted suicide [e.g. 1, 8–10] and advance directives [e.g. 11–14]. Policy governing assisted suicide and advance directives often introduces distinctions between psychiatric and non-psychiatric contexts. When it comes to assisted suicide, many jurisdictions that allow access to aid in dying do not extend such access to those whose suffering is primarily due to mental health – for example, Australia, some states in the United States, New Zealand, Austria, and Portugal. In the case of advance directives, treatment refusals tend to be binding, even life-limiting medical preferences, but most jurisdictions that recognize psychiatric advance directives allow clinicians to override expressed preferences in the case of involuntary psychiatric commitment – Germany being a notable exception (14). In light of principles of equality and non-discrimination, differential treatment between those with and without mental health conditions requires a strong justification. Jurisdictions that grant individuals with mental health conditions access to assisted suicide but limit the well-reasoned risk to self that such individuals can take on through use of an advance directive present particularly valuable cases to examine the principles underlying policy choices and evaluate the consistency of the application of those principles.

How should high-consequence decisions that intersect with clinical judgment be treated in psychiatric cases where the person’s preferences are well-considered and the context allows for decision-making capacity to be established? The following study explores this question by comparing policies regarding assisted suicide and advance directives in Switzerland, the Netherlands, and Germany, examining these countries’ approaches to handling psychiatric decision-making when it comes to respect for preferences with high consequences. The study aims to support well-founded legal and clinical practice with attention to potentially discriminatory practices.

## 2 Methods

Policies regarding assisted suicide and advance directives were compared in three different countries - Switzerland, Germany, and the Netherlands - with attention given to differences between the treatment of preferences primarily related to non-psychiatric conditions and the treatment of preferences primarily related to psychiatric conditions. These regions were chosen as all three do not fundamentally restrict access to assisted suicide for those with mental health conditions and all three legally recognize psychiatric advance directives but take significantly different policy approaches in ways that exemplify a range of framings (14). First, a comparison was made of policy on assisted suicide across the three jurisdictions. Then, a comparison was made of psychiatric advance directive policy across the three jurisdictions. Finally, a comparison was made between policy on assisted suicide and policy on psychiatric advance directives per jurisdiction.

## 3 Results

A brief overview of policy on assisted suicide and advance directives in Switzerland, the Netherlands, and Germany is presented in **Table 2** below.

**Table 2:** Brief policy summary.

Jurisdiction	Assisted suicide policy		Advance directive policy	
Switzerland	Both those with and without mental health conditions can, theoretically, access assisted suicide	Decriminalized with a reliance on clinical and ethical guidelines to set practice	Treatment refusals expressed in advance directives for non-psychiatric care are binding, even if such refusals may be life-limiting, but this is not always the case for psychiatric advance refusals	Clinicians can override preferences expressed in psychiatric advance directives if the person is involuntarily committed
The Netherlands		Under the purview of clinical care with a clear legal framework establishing practice		Same as above, although psychiatric advance directives are tailored to advance consent to treatment rather than advance refusals
Germany		A recent constitutional right for all, but lacking guidelines and established practices		There is full parity when it comes to respect for treatment refusals in both psychiatric and non-psychiatric cases

### 3.1 Assisted suicide policy

The following section lays out the regulation of assisted suicide in Switzerland, Germany, and the Netherlands with an emphasis on policy regarding access to assisted suicide in psychiatric cases.

#### 3.1.1 Assisted suicide policy in Switzerland

Assisted suicide has been decriminalized in Switzerland since 1942. Article 115 of the Swiss Penal Code stipulates that assisting another person to die is not punishable, provided the act is altruistic and free of selfish motives (15). Under this policy, access to assisted suicide in Switzerland is quite broad. In particular, there is no requirement for the individual to be terminally ill. Instead, the emphasis is on the individual’s capacity to make autonomous and informed decisions as well as the altruistic intentions of others involved. As such, the Swiss conception of permissible assisted suicide extends to individuals suffering from mental health conditions as long as those individuals can demonstrate the capacity to make autonomous and informed decisions.

Swiss law regarding assisted suicide does not lay out terms for specific procedural safeguards; instead, medical and ethical guidelines serve a crucial role in defining the practice and setting criteria that should be met. The process tends to follow the guidelines established by the Swiss Academy of Medical Sciences (SAMS), which have been adopted as part of the professional code of the Swiss Medical Association (Foederatio Medicorum Helveticorum) (16). These guidelines require the person to have decision-making capacity in relation to assisted suicide; the wish to die well-considered, enduring, and not due to external pressure; the symptoms of the condition to be a source of intolerable suffering; and other options to be ineffective or reasonably rejected by the person. There is no required physician involvement since the legal approach does not place assisted suicide within systems of clinical care, but medical professionals are often involved in evaluating the request for assisted suicide and providing the means to carry out the act. Euthanasia, the direct act of ending a person’s life by a third party, is illegal according to Article 114 of the Swiss Penal Code, which criminalizes “homicide at the request of the victim.” As such, the person seeking to end their life should perform the act itself (15). The Swiss model is unique in its minimal legal intervention, relying heavily on ethical and medical guidelines rather than codified safeguards.

#### 3.1.2 Assisted suicide policy in the Netherlands

Regulated by the Termination of Life on Request and Assisted Suicide Act of 2002, the Netherlands has a well-defined regulatory framework for both euthanasia and assisted suicide. Neither euthanasia nor assisted suicide is punishable if certain due care criteria are met (17). Aid in dying, understood as encompassing both euthanasia and assisted suicide, was first decriminalized in the Netherlands following case law, which recognized a conflict constituting “force majeure” between clinicians’ duty to preserve life and their duty to relieve suffering when confronted with patients who suffer severely and irremediably (1, 18). Given that clinicians uniquely face this professional ethical bind, aid in dying in the Netherlands is legally understood as a clinical act, decriminalized only if it is carried out by clinicians. As such, access to this aid is based not solely on the principle of autonomy, but also on the principle of beneficence and the clinicians’ role in relieving suffering (19).

Due to the institutional purview of aid in dying in the Netherlands, strict criteria must be met (20, 21): The request must be voluntary and well-considered; the patient must be well-informed; and the patient’s suffering must be unbearable, without prospect of improvement, and without reasonable alternatives available to address it. After the responsible physician has determined that all due care criteria for assistance in dying are met, an independent physician, specially trained and certified (SCEN physician), must see the person requesting aid in dying, make an assessment, and similarly conclude that all criteria are met (21). The act itself must be carried out according to medical standards. All cases of aid in dying are subject to retrospective review by the Regional Euthanasia Committees.

Established through case law, mental health conditions are recognized as a valid basis for aid in dying in the Netherlands: provided all criteria are met, access is available to individuals suffering from both physical and mental health conditions. Both in case law and in the Euthanasia Code, it is stated that physicians must exercise “great diligence” (*grote behoedzaamheid*) in their assessment of the due care criteria when the person’s request is based on suffering due to a mental health condition (21). The Euthanasia Code operationalizes this requirement of great diligence in the form of an evaluation by an independent psychiatrist in addition to the assessment by the SCEN physician (21).

#### 3.1.3 Assisted suicide policy in Germany

In February 2020, the German Federal Constitutional Court overturned Section 217 of the Penal Code, which had made organized forms of assisted suicide illegal. By declaring section 217 unconstitutional, the court established the right to a self-determined death, including both the freedom to take one’s own life and to seek assistance from third parties for such purpose (22). Euthanasia, though, remains a criminal offense according to Section 216 of the Penal Law. In Germany, the argument for assisted suicide is based on respect for autonomy, upheld so long as the person’s wish to die follows from their own free will (22).

A request for assisted suicide based on the person’s free will presupposes, according to the court, that the person has the ability to form a will while being “uninfluenced by an acute mental disorder;” is well-informed about the circumstances of the decision, which notably includes knowledge about alternatives to assisted suicide; is not subject to undue influence from another; and has an enduring and persistent wish to die (22). The court makes clear that assisted suicide should not be restricted to cases of terminal or incurable illness; while there is a requirement that the request not be influenced by an acute mental disorder, this does not categorically restrict access to assisted suicide for those with mental health conditions (1, 22). The court established that the right to a self‐determined death and the right to seek assistance from third parties are independent of the person’s motives. The person’s motives may not be judged based on “general values, religious precepts, societal norms for dealing with life and death, or considerations of objective rationality” (1, 22).

Thus, those seeking assisted suicide due to mental health conditions can potentially access assistance, so long as they have an enduring and persistent wish to die, the conditions of informed consent are met, and the decision is not influenced by an acute mental health condition. While the ruling established a constitutional right and decriminalized assisted suicide, it did not put in place a regulatory framework. No specific laws have yet been established to govern the practice.

#### 3.1.4 Summary of assisted suicide policy across the three jurisdictions

All three jurisdictions create conditions whereby those with mental health conditions can, theoretically, access assisted suicide. As such, in the arena of assisted suicide, these jurisdictions all allow those with mental health conditions to take on a high degree of risk of harm to self in cases of sound decision-making. The underpinning principles, though, range quite significantly. When it comes to assisted suicide, Switzerland emphasizes altruism, the Netherlands beneficence, and Germany autonomy. Switzerland and Germany recognize principles that apply to citizens, while the Netherlands recognizes a principle that applies to clinicians. The Swiss approach is based on decriminalization with a reliance on clinical and ethical guidelines; clinicians have a role to play, but assisted suicide is not a clinical act. The Dutch approach regulates assisted suicide from within the purview of clinical care and has the clearest framework establishing protected access for those with mental health conditions – albeit following additional safeguards. The German approach holds assisted suicide as a constitutional right for all, those with and without mental health conditions alike, but, as yet, lacks clear regulatory safeguards and established practices.

### 3.2 Psychiatric advance directive policy

The following section lays out the regulation of psychiatric advance directives in Switzerland, Germany, and the Netherlands, including information on the regulation of traditional, non-psychiatric advance directives for context.

#### 3.2.1 Psychiatric advance directive policy in Switzerland

Switzerland introduced binding advance directives through a modification of the civil code (Schweizerisches Zivilgesetzbuch, ZGB) in 2013. Under Swiss law, competent individuals can use advance directives to specify which medical treatments they consent to or refuse in the event of future incapacitation. They can also appoint representatives to discuss and decide treatment with clinicians (23). Importantly, the law stipulates that “physicians shall comply with the advance directive unless it violates statutory regulations or there are reasonable doubts about whether it reflects the patient’s free will or current wishes” (23). If a valid advance directive is not followed, the reasons must be documented in the patient’s medical record (23).

Outside the context of involuntary commitment, individuals can use advance directives to refuse treatment, even if such refusal might lead to self-harm, conflict with medical judgment, or place additional burdens on the healthcare team or family members. Advance directives in the Swiss context can be used to take on a high degree of risk to self, including choices that may be life limiting, but not, generally, in the case of psychiatric care where involuntary commitment creates conditions by which advance directive preferences can be overridden and harm-reducing interventions imposed upon the person (14, 23). Thus, psychiatry operates under a form of law that deviates from the standards applied in non-psychiatric clinical care.

In cases of involuntary commitment (*Fürsorgerische Unterbringung*), the legal status of advance directives is weaker and need only be “taken into account” (23). Instead of appointing a full representative, individuals under involuntary commitment can involve a trusted person for support (23). Advance directives in the context of involuntary commitment in Switzerland primarily serve to communicate treatment preferences, experiences, and values or consent in advance to coercive measures in case of future incapacity. The preferences expressed, though, are not binding. The diminished status of psychiatric advance directives does not apply in cases where an individual is voluntarily undergoing inpatient psychiatric treatment. Notably, the diminished status of advance directives in the case of involuntary commitment also does not apply to advance statements regarding non-psychiatric medical treatments that the same person has made (23).

#### 3.2.2 Psychiatric advance directive policy in the Netherlands

Binding advance directives for treatment refusals were introduced in the Netherlands in 1995 when the Medical Treatment Agreement Act (WGBO) took effect. The WGBO is part of the Civil Code and applies to all Dutch citizens, regardless of whether they have a physical or mental health condition. Psychiatric advance directives, though, are regulated separately under the Mental Healthcare Act (*Wet verplichte geestelijke gezonheidszorg*, Wvggz), which is part of public law and regulates compulsory mental healthcare for people with a mental health condition. While people with mental health conditions can refuse treatment with an advance directive under WBGO, the Wvggz applies when a person has a mental health condition and poses a risk of harm to self or others (*ernstig nadeel*). The person can then be treated against objections and against preferences outlined in the advance directive if the legal criteria for involuntary treatment under the Wvggz are met.

Since 2008, psychiatric advance directives in the Netherlands have been tailored to self-binding treatment requests where the person consents to specific treatments in advance, making the Dutch legal framework for psychiatric advance directives notably distinct. Psychiatric advance directives in the Netherlands – known as self-binding directives – have clearly regulated requirements that differ from traditional advance directives (14, 24).

For example, at the time of creating the document, capacity must be assessed for psychiatric advance directives (25) but not for other advance directives. While no independent authorization is required to validate treatment according to traditional advance directives, involuntary treatment based on a self-binding directive must be legally authorized by a judge and include the review of a second, independent psychiatrist (25). While there are no content requirements for other advance directives, psychiatric advance directives must specify the conditions under which involuntary treatment should be provided; the kind of care requested; the duration of involuntary treatment; the period of validity of the document; and a contact person (25). The user must also set activation criteria for the psychiatric advance directive, indicating when the document should go into effect. To revoke or amend a psychiatric advance directive, the same conditions that apply to the creation of the document must be met (25). Much of the increased regulation around psychiatric advance directives in the Netherlands relates to the unique role these documents can play in binding someone to advance consent to treatment: the regulations both create safeguards and enable such documents to serve their intended function (14, 26).

#### 3.2.3 Psychiatric advance directive policy in Germany

In Germany, advance directives have been legally recognized since 2009 and regulated under guardianship law, specifically Sec. 1827 of the Civil Code (BGB) (27). Guardianship law applies to all people who need support in managing their own affairs, regardless of whether they have a mental health condition or not (28). Parliamentary discussions leading up to the legislation on advance directives, the provisions of Sec. 1827 of the Civil Code, made clear that the statute applies to physical and mental health conditions alike. The same holds for the sections on involuntary commitment (Sec. 1831 BGB) and involuntary treatment (Sec. 1832 BGB). As such, there is no separate federal legislation for advance directives in psychiatric contexts.

In Germany, people can use an advance directive to express treatment preferences or to appoint a legally authorized representative. There are no content requirements for advance directives except that the preferences and the circumstances in which they should apply be described specifically. Treatment refusals are legally binding. Since compliance with the person’s advance directive is a necessary condition for involuntary treatment (Sec. 1832 para 1 no 3), treatment refusals outlined in an advance directive must be respected even if the person is under involuntary commitment and even if complying with the treatment refusal is not in the person’s clinical best interests (27). Advance directives can only be overridden if there are well-grounded reasons to doubt the validity or applicability of the document. Involuntary commitment or coercive measures to prevent harm to others cannot be refused by means of an advance directive, but the person cannot then be treated against the preferences outlined in their advance directive. There are no formal requirements to amend or revoke the document, which makes sense in light of the high risk of harm to self that one is allowed to assume through advance treatment refusal and the relatively low risk of allowing that person to easily overturn their own directive and, instead, consent to care (14).

#### 3.2.4 Summary of psychiatric advance directive policy across the three jurisdictions

In all three jurisdictions, treatment refusals for non-psychiatric care are binding, even if such refusals may be life-limiting, but the three jurisdictions handle the risk a person can assume in psychiatric cases quite differently from one another. Swiss policy establishes means whereby clinicians can override well-considered preferences expressed in psychiatric advance directives if the person meets the criteria for involuntary commitment, which does not require a lack of mental capacity; this limits the person’s self-determination in a way that clearly differs from non-psychiatric cases. Similarly, the Dutch system does not allow people with mental health conditions to take on the same degree of high-risk refusals that they do for those using advance directives in non-psychiatric cases; in the Netherlands as well, preferences in advance directives can be overridden in cases of involuntary treatment. The distinct regulations in the Netherlands establish psychiatric advance directives as a tool to aid clinical intervention by facilitating advance consent to care. The status of Dutch psychiatric advance directives is, therefore, distinct from both traditional advance directives in the Netherlands and psychiatric advance directives in Germany and Switzerland. Germany is the only of the three jurisdictions with full parity between the risk to self that one with decision-making capacity can assume through advance directives in psychiatric and non-psychiatric cases. Involuntary commitment that goes against preferences in an advance directive is possible in Germany only in cases where the person is a potential harm to others. In the case of Germany, involuntary commitment does not legitimize treating the person against preferences expressed in their advance directive.

### 3.3 Comparison of assisted suicide policy and psychiatric advance directive policy per jurisdiction

The following section draws comparisons between assisted suicide policy for those with mental health conditions and psychiatric advance directive policy in each jurisdiction separately, evaluating consistency between the two policies.

#### 3.3.1 Comparison of policy for assisted suicide in psychiatric cases and policy for psychiatric advance directives in Switzerland

With demonstrated decision-making capacity, people with mental health conditions in Switzerland can, theoretically, take on a high degree of life-limiting risk in the case of access to assisted suicide but not in the case of advance refusal of psychiatric interventions. In both cases, there is an absence of codified, recognized rights for the specific practices. Swiss regulations are consistent in deference to clinician judgment that is shaped by ethical and clinical guidelines. Clinicians have a high degree of freedom to follow their discretion and submit people to clinical best judgment.

#### 3.3.2 Comparison of policy for assisted suicide in psychiatric cases and policy for psychiatric advance directives in the Netherlands

The Netherlands is unique in that neither aid in dying nor psychiatric advance directives gives people a right based on autonomy to make potentially life-limiting choices themselves. In the case of both aid in dying for those with mental health conditions and psychiatric advance directives, the Dutch context is coherent in its emphasis on beneficence and the role of clinical institutions. The focus of the law puts power in the hands of clinicians to provide care they deem to be in the person’s best interest and in accordance with the person’s choices. In the case of aid in dying, this does sometimes translate to life-limiting interventions. Psychiatric advance directives, on the other hand, are designed to facilitate interventions to preserve life and mitigate possible harm to self.

#### 3.3.3 Comparison of policy for assisted suicide in psychiatric cases and policy for psychiatric advance directives in Germany

In theory, people with mental health conditions in Germany can take on a high degree of risk in the case of both assisted suicide and advance directives. The applicable standards are not substantially different for those with and without mental health conditions. Although Germany has established rights based on autonomy, there is continued need for regulation to define the practices. In the case of assisted suicide, the right is relatively newly introduced, and a legal framework is not yet in place. In the case of advance directives, the regulation introduces new scenarios that require new practices; for example, how to handle cases of involuntary commitment when the person has refused treatment. Germany takes a clear position with consistent respect for autonomy, but regulatory guidelines to support the rulings and guide clinicians are still needed.

## 4 Discussion

In the above comparison, three different pictures emerge concerning people’s right to have well-considered, high-consequence preferences respected in psychiatric cases. For this discussion, the jurisdictions can be differentiated by 1) a high degree of deference to clinician judgment in the case of Switzerland, 2) arguments founded on the clinician’s duties to patients in the case of the Netherlands, and 3) recognition of inviolable rights that apply uniformly to all in the case of Germany. Implicit in an examination of respect for well-considered, high-consequence decisions is the issue of coercion. In that regard, the three different positions of the countries have significantly different implications for practice.

The Swiss context tends toward clinician discretion; access to assisted suicide and the use of psychiatric advance directives to express binding treatment refusals are not legal rights. Data suggest a readiness for clinicians to exert influence in high-consequence decision-making. In 2022, Switzerland recorded at least 18,367 cases of involuntary commitment (29). Those rates, some of the highest in Europe, have been increasing and continue to rise (30). The proportion of involuntary commitment among all psychiatric cases varies by canton, ranging from 10% to nearly 40% (31). These rates can be considered alongside the fact that self-determined dying is not framed as an autonomy-based right nor are psychiatric advance directives imbued with the legal force to protect autonomous decision-making in the face of involuntary commitment. Policy choices that do not codify such autonomy-based rights may inform a system in which clinicians have strong influence over those with mental health conditions in instances of high-consequence decision-making and a tendency to impose clinical best judgment.

The Dutch context recognizes a large and central role for clinicians in high-consequence decision-making, but that clinical power exists alongside a strong legal framework establishing psychiatric patients’ right to have well-considered decisions respected. Aid in dying and psychiatric advance directives policy are both organized around legally facilitating the clinician’s duty to care for patients even if the interventions are high-consequence – euthanasia in the case of aid in dying and binding someone to their previously stated preferences for treatment in the case of psychiatric advance directives. This is an approach that points toward decoupling beneficence from possible coercion: as the legal support for high-consequence clinical interventions based on beneficence is strengthened, so too is the patient’s role and right to express well-considered preferences. The legal power physicians assume in the name of beneficence is toward the enactment of the person’s wishes. While coercion in Dutch mental healthcare steadily increased following the introduction of the Special Admissions to Psychiatric Hospitals Act (Bopz) in 1994, data from the national Council for the Judiciary (Rvdr) shows that the upward trend lessened with the introduction of the Wvggz in 2020 (32). While the Dutch system codifies a strong role for clinicians and institutions, bestowing upon them considerable control, the power is not, primarily, a means to override people’s preferences paternalistically but rather to facilitate the execution of high-consequence preferences.

The German context has the greatest emphasis on recognized rights and legally articulated parity in cases of mental illness, most clearly limiting the role of clinicians as arbiters of well-considered, high-consequence decisions. Since there is no nationwide, systematic recording of the frequency of involuntary commitment and coercive measures in Germany, the trends are difficult to gauge, but psychiatric advance directives offer a definitive means by which people can express binding, advance refusals of treatment. Policy in Germany aims strongly toward protecting the agency of individuals and providing means by which well-considered, high-consequence decisions have legal support. The person’s right to a self-determined death is independent of their motives and refusals articulated in valid psychiatric advance directives stand even in the face of involuntary commitment. While the person’s rights are clear, how clinicians and institutions should best interact with those rights is less clear. The strong rights codified in law introduce unprecedented clinical situations requiring new approaches. Further regulatory structures and guidelines are needed, for example, to indicate how clinicians should engage with requests for assistance in dying or how they should engage with someone who has been involuntarily hospitalized as a threat to others but who has an advance statement refusing the treatment that would lead to rehabilitation and allow for hospital discharge (33). A clear defense of the rights of those with mental health conditions to take on well-considered, high-consequence decisions still requires a framework to guide clinicians in a system that firmly limits paternalism.

The question this comparison raises is how policy should guide clinical institutions and clinicians to interact with well-considered decisions that have significant health-related implications. Such cases highlight the tension between beneficence and autonomy since these well-considered preferences may lead to harm. As this review demonstrates, there is a role for institutions and clinicians, but that role needs further reflection. There is special attention required when it comes to psychiatric cases given the way policy presently creates distinctions, or, in moving away from distinctions, new challenges. Policy around assisted suicide, advance directives, and coercion are interrelated since all three aim to properly balance respect for autonomy with clinician’s duties; there is value in considering them together.

Some researchers have raised the question of whether greater deference to patient autonomy in psychiatric care and even, potentially, palliative approaches that emphasize quality of life over preservation of life might significantly reduce suffering as well as recourse to assisted suicide (34–38). Where the harm of coercion is mitigated and people have options for care even in the face of treatment futility, assisted suicide may be less compelling. Since advance directives are a tool to align care with patients’ goals and mitigate paternalism the person may perceive as harmful, advance directives with strong legal force may have a role to play in reducing recourse to assisted suicide to the extent that they help users assert autonomy, preserve dignity, or lessen iatrogenic harm in psychiatric settings.

On the other hand, advance directives have come under scrutiny as possible means to facilitate suicide (39–41). In some jurisdictions, this concern may be a factor in weakening the legal force of advance directives in psychiatric care (14). While evidence suggests that psychiatric advance directives are not used for general treatment refusals (42, 43), policy around the rights of those with mental health conditions to seek assisted suicide may inform the question of what degree and what type of risk someone can assume through well-founded advance refusals of psychiatric interventions. Sound and consistent practice when it comes to both assisted suicide and advance directives is needed in order to fairly respect the rights of those with mental health conditions and to move toward models that truly best address suffering.

## 5 Conclusion

In psychiatric care, high-consequence decisions often arise in emergency contexts where it can be hard to establish if the patient’s decision-making is sound and easier to justify applying clinical best judgment, but that is not the case for either formal assisted suicide requests nor preferences expressed in valid advance directives. As such, carefully constructing policy around assisted suicide and advance directives can help properly define the relationship between institutions and people’s high-consequence preferences that have been well-considered. In pluralist secular societies, self-determination is a strong guiding principle that calls for respect of people’s preferences, even respect for choices that put health at risk or shorten life. The picture is complicated, though, when those choices intersect with or depend upon clinical institutions and clinicians, who themselves carry responsibilities and agendas.

Comparative studies like the above help elucidate a range of possible approaches and the implications of those different approaches. The Swiss Academy of Medical Sciences is currently revisiting its guidelines on coercion (44), which is an opportunity to reconsider whether underlying values are being consistently applied and having their intended effects on practice. The present comparative study suggests possible directions. For example, the Swiss context may benefit from more formally recognizing the rights of individuals, as Germany and the Netherlands do. Germany will likely benefit from establishing guidelines that structure practice; such guidelines are central in both Swiss and Dutch practice. The Dutch context may benefit from exploring the relationship between upholding advance refusals of psychiatric care – which is more so the emphasis in Switzerland and Germany - and people’s experiences of suffering.

In general, we hope the present study might inspire countries to critically review policies that define respect for well-considered, high-consequence decisions with an eye to the consistent application of guiding principles, awareness of the implications of those principles, and special care to avoid promoting unjustified differential treatment of those with mental health conditions.

## Glossary

Assisted suicide: Aiding a person in the process of ending their own life by providing the means to do so
Euthanasia: The direct act of ending a person’s life by a third party
Aid in dying: An umbrella term that encompasses both euthanasia and assisted suicide
Advance directives: Documents used to declare treatment preferences in the event of future loss of decision-making capacity
Psychiatric advance directives: Advance directives that specifically relate to psychiatric care
Decision-making capacity: A person’s ability to understand, appreciate, reason, and express their preferences in relation to a specific treatment decision
Involuntary commitmen: Admitting a person to clinical facility without the person’s consent
Involuntary treatment: Carrying out medical or therapeutic interventions without the person’s consent
Coercion: Measure applied against a person’s will and/or in spite of their opposition
